# Interventions to reduce emergency department overcrowding and their effects on patient outcomes: a mixed-methods systematic review and meta-analysis

**DOI:** 10.1186/s12873-026-01587-8

**Published:** 2026-04-25

**Authors:** Weaam Alamri, Farah Aljuhani, Nouf Alsharef, Rebal Khayyat, Manal Shuaib, Tahira Faheem, Abdu Alsayed

**Affiliations:** 1 Department of Medicine and Surgery, Batterjee Medical College, Jeddah, 21442 Saudi Arabia; 2 Department of Emergency Medicine, Ministry of National Guard Health Affairs, Jeddah, Saudi Arabia

**Keywords:** Emergency Department (ED), Overcrowding, Length of stay (LOS), Fast track, Team triage, Point Of Care Testing (POCT)

## Abstract

**Background:**

Overcrowding in emergency departments (EDs) leads to inadequate patient outcomes and increased operational costs. These complications contribute to heightened stress on healthcare systems thus, effective methods to reduce ED overcrowding are necessary to improve patient flow and overall care quality.

**Aim:**

This mixed-method systematic review and meta-analysis evaluates the effectiveness of interventions such as Fast-Track, Team Triage, and Point-Of-Care Testing (POCT) in reducing ED overcrowding and improving patient flow metrics, including ED length of stay (LOS), wait times, and discharge times, to provide evidence-based recommendations for policy and practice in emergency healthcare.

**Methods:**

This review complies with the Preferred Reporting Items for Systematic Reviews and Meta-Analyses (PRISMA) guidelines. Three databases (PubMed, ScienceDirect and Google Scholar) were systematically searched for studies published between 2015 and 2025 assessing interventions to reduce ED overcrowding. A structured risk-of-bias assessment (RoB 2, ROBINS-I, JBI, MMAT) indicated overall acceptable methodological quality, with expected limitations related to non-randomized designs and limited blinding. Mean LOS was pooled using random-effects meta-analysis in RevMan, with heterogeneity assessed and secondary outcomes summarized narratively. This study is registered with the International Prospective Register of Systematic Reviews (PROSPERO 2025: CRD42025634351).

**Results:**

This review included 15 studies, 6 eligible for meta-analysis. Fast-Track significantly reduced ED length of stay (MD = − 20.89 min; 95% CI: − 37.05 to − 4.33; *p* = 0.01). POCT (MD = − 65.50 min; 95% CI: − 137.54 to 6.53; *p* = 0.07) and Team Triage (MD = − 46.11 min; 95% CI: − 120.59 to 28.38; *p* = 0.23) demonstrated variable reductions in ED LOS, with pooled effects influenced by substantial between-study heterogeneity. All analyses exhibited significant heterogeneity. Qualitative synthesis further suggested improvements in waiting times, patient throughput, and departmental efficiency.

**Conclusion:**

Our findings highlight the importance of targeted interventions to alleviate ED overcrowding; Fast-Track showed consistent effectiveness, while POCT and Team Triage showed variable and, often context-dependent effects. These findings offer practical and evidence-based strategies to support ED flow improvements, though further high-quality research is needed.

**Supplementary Information:**

The online version contains supplementary material available at 10.1186/s12873-026-01587-8.

## Introduction

 Overcrowding in Emergency Departments (ED) has emerged as a critical challenge for public health systems globally over the past decades [1]. The demand for emergency care, even in the most developed countries, has increased considerably due to longer life expectancy and the resulting rise in chronic degenerative diseases [2]. However, the expansion of healthcare resources has not kept pace with these growing demands. This imbalance has made it increasingly difficult for hospital emergency services to fulfill their primary mission of providing timely and high-quality care. Typical scenes in overcrowded EDs include fully occupied beds, patients placed in hallways, and overcrowded waiting rooms where individuals may wait for hours [3, 4]. Healthcare staff, meanwhile, face substantial pressure and stress [5]. The consequences are significant and include patients leaving without receiving care, ambulance diversions, delayed access to medical services, prolonged lengths of stay (LOS) in EDs and hospitals, increased risk of iatrogenesis and treatment delays, slower recovery times, higher rates of morbidity and mortality, escalating operational costs, and reduced patient satisfaction [3].

Multiple strategies have been introduced to reduce overcrowding, particularly those aimed at improving internal ED processes and accelerating clinical decision-making. Common interventions include Fast-Track systems, Team Triage, and Point-of-Care Testing (POCT). Fast-Track systems accelerate treatment for less acute patients, which may reduce overall LOS and waiting times. Team Triage adopts a collaborative approach to the initial patient assessment, aiming to enhance efficiency and patient flow. POCT enables immediate bedside diagnostic testing, reducing diagnostic turnaround time (TAT) and potentially improving throughput. Although these interventions have been widely implemented in EDs, their comparative effectiveness in improving key operational outcomes remains an area of ongoing research. Although several studies have examined interventions to improve ED flow, few systematic reviews have simultaneously evaluated Fast-Track systems, Team Triage, and POCT using both quantitative meta-analysis and qualitative synthesis.

This study adopts a PICO framework. The Population includes general ED patients regardless of demographics or presenting conditions. The Interventions involve Fast-Track systems, Team Triage, and POCT. The Comparator is standard ED care without these targeted interventions. The Outcomes of interest are operational metrics related to ED patient flow: ED LOS, defined as the time from patient arrival until final disposition; Wait Time, defined as the interval between ED registration and the initial provider assessment; and Time to Triage, which refers to the time between patient arrival and completion of formal triage.

This study aims to systematically synthesize evidence on the effectiveness of these commonly implemented ED interventions in reducing overcrowding and improving patient flow. By evaluating their impact on LOS, waiting times, and time to triage, the findings aim to provide evidence-based insights to inform policy and clinical practice in emergency healthcare settings.

## Methods

### Ethics and registration

This systematic review is registered in PROSPERO under the (registration number CRD42025634351). Ethical approval was not required as no primary data was collected. All included studies were published in peer-reviewed journals, and adherence to ethical standards by the original investigators is assumed. The review was conducted in accordance with PRISMA guidelines to ensure transparency, rigor, and integrity throughout study selection, quality assessment, and synthesis.

### Study design

This systematic review adhered to the Preferred Reporting Items for Systematic Reviews and Meta-Analyses (PRISMA) guidelines to ensure methodological rigor and transparency. Using a mixed-methods approach, it systematically identified and synthesized both quantitative and qualitative studies evaluating interventions implemented in EDs and assessed their impact on overcrowding, wait times, and patient outcomes.

### Search strategy

A comprehensive literature search was conducted in PubMed, Google Scholar, and ScienceDirect for studies published between 2015 and 2025. Searches were limited to English-language publications. To ensure both breadth and precision, a combination of Medical Subject Headings (MeSH) and free-text keywords was used. Search terms were organized around the main concepts of the review, including the emergency department setting, intervention types (Fast-Track, Team Triage, point-of-care testing [POCT], and clinical decision support systems [CDSS]), and outcomes related to wait times and length of stay (LOS).

In PubMed, MeSH terms were combined with synonyms and keyword variants using Boolean operators (AND/OR). The complete search strategies for all databases, including the full search strings used in PubMed, Google Scholar, and ScienceDirect, are provided in Supplementary Table S1.

The search strategy was initially developed and implemented by one reviewer using MeSH terms and keyword combinations, and a second reviewer verified the search results for completeness.

### Eligibility criteria

The eligibility criteria for this systematic review were defined using the PICOS framework. Studies were included if they focused on hospital-based ED patients, evaluated interventions such as Fast-Track systems, Team Triage, or POCT compared to standard care, and reported patient flow metrics like LOS, wait times, time to triage, and discharge times. Eligible studies were original research, including randomized controlled trials, cohort studies, case-control studies, and simulation studies evaluating interventions to reduce ED overcrowding, published in peer-reviewed journals in English between 2015 and 2025. Exclusion criteria encompassed studies unrelated to ED overcrowding interventions, those not reporting patient outcomes, non-English articles without translations, articles published before 2015, and review articles, commentaries, or editorials.

### Selective strategy

The study selection process involved three reviewers who independently screened titles and abstracts using the Rayyan platform to determine relevance to the research question. Each study was assessed by all reviewers, and disagreements were resolved through group discussion.

For full-text screening, four independent reviewers evaluated all eligible articles to determine whether they met the predefined inclusion and exclusion criteria. Any disagreements were discussed collectively, and final decisions were reached through consensus.

Although Clinical Decision Support Systems (CDSS) were included in the initial search strategy, studies evaluating CDSS were ultimately excluded because they did not meet the predefined eligibility criteria.

### Data extraction

Data from all included studies were systematically extracted using a standardized Excel data extraction sheet. Extracted variables included author, publication year, country, study design, clinical setting, and population characteristics such as sample size and eligibility criteria.

Details regarding the interventions (e.g., POCT, Team Triage, Fast-Track systems) and comparator conditions were documented, along with intervention duration when available. The primary outcome was ED length of stay (LOS), while secondary outcomes included wait time, triage-to-provider time, and discharge time.

For clarity, “wait time” was defined as the interval from ED registration to initial provider assessment, while “door-to-provider time” refers to the time from patient arrival to first contact with a healthcare provider. These outcomes were treated as distinct measures where reported.

Statistical measures such as means, medians, interquartile ranges (IQRs), p-values, and confidence intervals were also extracted. Contextual information, including funding sources and study limitations, was recorded.

Two independent reviewers conducted the data extraction to ensure accuracy and minimize bias. For studies reporting multiple time points, the most comparable and commonly reported outcomes were selected using predefined decision rules.

**Table 1:** Outcome definitions and harmonization across included studies

Study	Reported outcome	Original definition	Standardized category
Chrusciel et al. 2019	ED LOS	Time from ED registration to patient leaving the ED	ED LOS
Copeland et al. 2015	Wait time	Time from triage to first physician assessment (inferred)	Wait time
	ED LOS	Time from triage to disposition (inferred)	ED LOS
Hausfater et al. 2020	ED LOS	Time from administrative registration to medical decision (discharge/admission)	ED LOS
	Wait time	Time from registration to first medical contact	Wait time
Chartier et al. 2015	Physician initial assessment time	Time from triage to first physician (or delegate) assessment	Wait time
	ED LOS	Time from triage to ED discharge	ED LOS
Van der Linden et al. 2019	ED LOS	Not explicitly defined*	ED LOS
Van der Linden et al. 2024	Triage wait time	Time from registration to triage level assignment	Time to triage
	ED LOS	Time from registration to departure	ED LOS
Kuo et al. 2018	Patient waiting time	Not explicitly defined (simulation-based delay before care)	Wait time
Ross et al. 2024	Door-to-provider time	Not explicitly defined	Wait time
	ED LOS	Not explicitly defined	ED LOS
Peng et al. 2020	ED LOS	Not explicitly defined (modeled)	ED LOS
	Wait time	Not explicitly defined (modeled)	Wait time
Chaves et al. 2022	ED LOS	Not explicitly defined	ED LOS
Goldstein et al. 2018	Throughput time	Includes front-end time, time to physician and treatment time	ED LOS
McAvoy et al. 2020	Acute bed occupancy	Not explicitly defined (model-derived)	Other (crowding metric)**
	Ambulance ramping	Not explicitly defined (model-derived)	Other (crowding metric)
Kaushik et al. 2018	ED LOS	Time from ED admission to discharge	ED LOS
Ienghong et al. 2021	ED LOS	Time from triage registration to discharge order in electronic medical records (EMR)	ED LOS
Duma et al. 2023	ED LOS	Not explicitly defined	ED LOS
	Door-to-provider time	Not explicitly defined	Wait time

NOTES: *In studies where outcomes definitions were not explicitly provided, standard ED operational definitions were assumed based on commonly accepted clinical workflow) (**Additional outcomes (e.g. laboratory turnaround time and crowding metrics) were identified but excluded from harmonization as they did not align with predefined endpoints

### Quality assessment

Study quality was assessed using validated tools according to study design. The RoB 2 tool was applied to randomized trials, while ROBINS-I was used for non-randomized studies. Cross-sectional studies were evaluated using the JBI Critical Appraisal Checklist, and qualitative studies were assessed using the JBI Qualitative Checklist. Mixed-methods studies were appraised using the Mixed Methods Appraisal Tool (MMAT).

These tools evaluated factors including participant selection, measurement validity, intervention classification, confounding, and ethical considerations. Two reviewers independently conducted the quality assessments.

Inter-rater agreement was high (κ = 0.89). One disagreement was resolved through discussion, resulting in a final classification of high risk of bias for that study.

### Data synthesis

Due to the variability between outcome definitions across the included studies, a harmonization approach was applied as summarized in Table 1. Studies that reported conceptually similar time-based metrics were grouped under standardized ED categories (e.g. wait time, ED LOS, time to triage). These outcomes were included if they reflected comparable clinical processes within standard ED flow.

Study results were summarized in tables highlighting characteristics, interventions, and outcomes. Meta-analyses were conducted using RevMan Web to compare mean ED LOS across Fast-Track, Team Triage, and POCT interventions versus controls. The values of means and standard deviations were directly extracted from studies that reported them. For studies reporting medians, the medians were assumed to be equal to means due to the absence of distributional data. This method is frequently used in meta-analyses for LOS when conversion is not practical in accordance with the Cochrane Handbook. If not available, standard deviations were calculated from IQRs using approximation formulas (SD ≈ IQR/1.35) [6]. These approaches were used for inclusion of all relevant studies.

Mean difference (MD) with 95% confidence intervals was used as the effect size. A random-effects model was used due to high heterogeneity. Contributing factors included patient demographics, acuity, baseline LOS, and variations in intervention implementation. Sensitivity analyses and subgroup evaluations were conducted, with outliers removed where necessary.

Secondary outcomes, including wait times, triage-to-provider time, and discharge time, were analyzed thematically by intervention type. Comparisons between interventions and controls were made, and contextual factors influencing effectiveness were noted. Risk of bias was considered in interpreting these findings.

### Standard emergency department care

Standard ED care follows a structured framework involving triage, physician evaluation, diagnostics, and patient disposition. Emergency Severity Index (ESI) and Manchester Triage System (MTS) are triage systems that prioritize high-acuity cases, leading to prolonged wait times for lower-acuity patients [7]. Additional factors, including the need for prior physician evaluation before ordering labs and radiology, limited staff, bed shortages, and TAT for investigation results, can further delay the process [8, 9]. Standard ED care generally involves lengthy processes, which interventions aim to optimize to improve wait times, LOS, and overall patient throughput [10–12].

### Qualitative comparison

Fast-Track systems aim to improve ED efficiency by directing low-acuity patients to separate treatment pathways. This approach reduces congestion in the main ED and allows higher-acuity patients to receive timely care. By streamlining patient flow and optimizing resource allocation, Fast-Track models may reduce ED LOS and improve overall efficiency, particularly during peak periods.

Team Triage models, including Physician-in-Triage (PIT), enhance the initial assessment stage by involving senior clinicians early in the triage process. Unlike traditional nurse-led triage, PIT enables earlier diagnostic decision-making and test ordering, potentially reducing patient bottlenecks and improving workflow efficiency.

POCT accelerates diagnostic processes by providing rapid bedside test results and reducing reliance on centralized laboratory services. In standard ED settings, laboratory tests are typically ordered after physician evaluation and may require extended processing times. POCT enables faster diagnostic decision-making and may contribute to shorter patient waiting times and improved departmental efficiency.

However, the magnitude and consistency of these improvements varied across studies and healthcare settings, particularly for POCT and Team Triage interventions.

### Quantitative comparison

The included studies demonstrated measurable improvements in ED workflow compared with standard care. Overall, interventions were associated with reductions in waiting times and improved patient flow metrics.

Fast-Track systems demonstrated the most consistent improvements, with an average reduction of 13.82 min in waiting time across studies [11, 12]. Team Triage interventions, including PIT models, reduced waiting times by approximately 8.02 min by enabling earlier physician assessment and diagnostic ordering [8, 9, 13]. POCT interventions resulted in an average reduction of 7.12 min, primarily by accelerating diagnostic workflows [9, 14].

Overall, Fast-Track interventions showed the most consistent evidence of improving ED efficiency, reducing waiting times, and optimizing patient flow compared with standard ED care. Evidence regarding POCT and Team Triage remained more heterogeneous across different study settings.

## Results

**Fig. 1:**
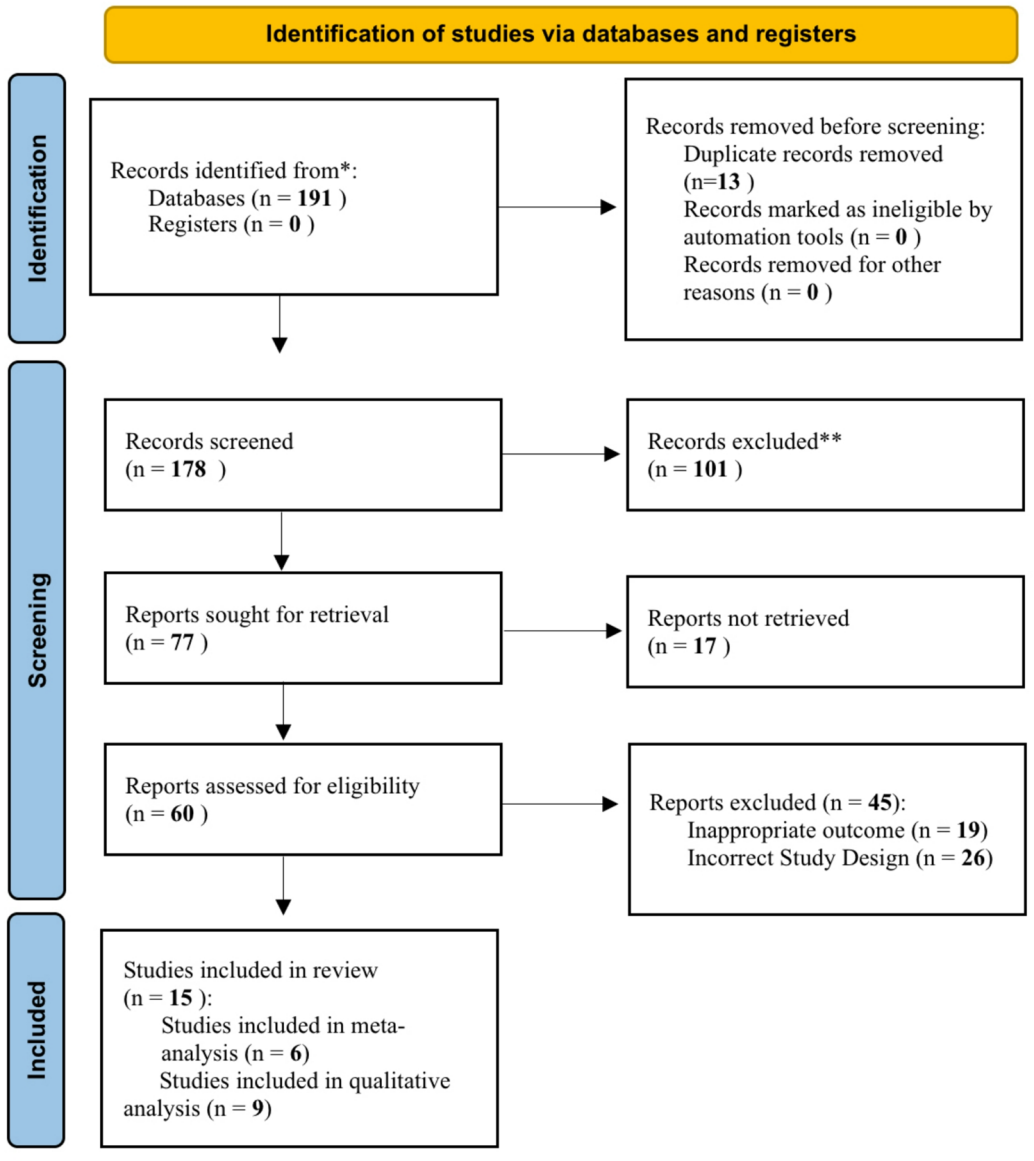
Identification of studies via databases and registers

### Study selections

The PRISMA 2020 flow diagram, shown in Fig. 1, shows each phase of the research identification and selection process, including the number of records identified, screened, excluded, and eventually included in the qualitative and quantitative analyses. The diagram visually maps how the studies progressed through the stages of database searching, duplicate removal, title and abstract screening, full-text assessment, and final inclusion or exclusion, providing a clear overview of the review process. A total of 191 records were identified through database searching. After removing 13 duplicate records, 178 records remained for screening. Following title and abstract screening, 101 records were excluded, and 77 full-text reports were sought for retrieval. Of these, 17 reports could not be retrieved.

The remaining 60 reports were assessed for eligibility. After full-text review, 45 studies were excluded due to inappropriate outcomes (*n* = 19) or incorrect study design (*n* = 26). Ultimately, 15 studies met the inclusion criteria and were included in the systematic review. Of these, 6 studies were eligible for meta-analysis, while 9 were included in the qualitative synthesis only.

**Table 2:** Characteristics of included studies, interventions, and key outcomes (all time-based measures reported in minutes)

Study ID	Year	Country	Study design	Sample size	ER Type	Intervention	Comparator	Outcome measured	Results summary
Chrusciel et al. 2019	2019	France	Retrospective before–after	*n* = 111,733	Adult ED	Fast-track implementation	Pre-intervention	ED LOS ≥ 4 h, access block, LWBS, 30-day readmission	LOS reduced (215→186 min); LWBS 10%→5.4%; readmissions 12.3%→11.4%; reduced access block
Copeland et al. 2015	2015	Canada	Retrospective before–after	*n* = 50,867 total (24,977 pre; 25,890 post)	Mixed (Adult + Pediatric)	Daytime fast-track for CTAS 4–5 patients	Pre-intervention	Wait time, LOS, CTAS compliance, LWBS	Wait time − 6 min; LOS − 15 min; CTAS-4 wait − 13 min; improved compliance; no change in LWBS
Hausfater et al. 2020	2020	France	Cluster randomized controlled trial	4 hospitals (~ 69,000 visits/year)	Adult ED	POCT	Central laboratory testing	Time to results, LOS	Time-to-result reduced (~ 51 min); LOS reduction small (− 9 min); POCT subgroup − 17 min; cost saving €31/hr
Chartier et al. 2015	2015	Canada	Quality improvement before–after	*n* = 1,509 total (755 pre; 754 post)	Mixed	Team triage (Rapid Medical Evaluation unit)	Usual care	Physician Initial Assessment, LOS	Assessment time 98→70 min; LOS 165→130 min for CTAS 4–5
Van der Linden et al. 2019	2019	Netherlands	Quality improvement before–after	*n* = 64,012 total (31,891 pre; 32,121 intervention)	Mixed	ENPs + specialists at peak hours, Lean radiology, extended admissions	Pre-intervention period	Crowding, radiology TAT, LOS, LWBS, return visits	Crowding 18.6%→3.5%; radiology TAT 91→50 min; LOS 167→154 min; reduced LWBS and returns
Van der Linden et al. 2024	2024	Netherlands	Quality improvement before–after	*n* = 3,562 total (1,330 intervention; 2,232 control)	Mixed	Triage station, CRP POCT, surgeon redirect, second waiting room	Control weeks	Triage wait time, LOS, crowding	Triage wait − 26%; LOS 184→168 min; reduced referral LOS
Kuo et al. 2018	2018	Hong Kong	Discrete-event simulation	N/A	Adult ED	Fast-track system	Standard workflow	Waiting time, congestion, physician utilization	Reduced overall waiting time; slight increase for higher-acuity patients
Ross et al. 2024	2024	USA	Quality improvement before–after	N/A	Pediatric urgent care	Fast-track for low-acuity pediatric patients	Standard workflow	Door-to-provider time, LOS, LWBS	Reduced door-to-provider time and LOS; no adverse impact on higher acuity
Peng et al. 2020	2020	Canada	Discrete-event simulation	39,525 records	General ED	PIA	Nurse triage	LOS, Wait time	LOS − 34%; waiting time − 49%; optimal during peak hours
Chaves et al., 2022	2022	Brazil	Qualitative, descriptive	N/A	Emergency Care Units	Fast Track method	None	LOS, workflow efficiency	Reduced LOS for low-acuity patients; improved workflow and safety
Goldstein et al. 2018	2018	South Africa	Randomized controlled trial	1044	Adult ED patients	POT	Standard workflow	Treatment time	Significant reduction in treatment time; no benefit for psychiatric patients
McAvoy S et al., 2020	2020	Australia	System dynamics modeling	approx. 2,500	Tertiary ED	Scenarios: extra beds, short-stay beds, doctors	Base case	Bed occupancy, ambulance ramping	Optimal flow requires combined interventions; single changes limited effect
Kaushik et al., 2018	2018	USA	Retrospective EHR analysis	Multisite: 463,712 visits; Single-site: 11,247 visits	Adult ED	Reduced lab TAT	Standard TAT	LOS, admissions	1-min TAT reduction → ~0.5 min LOS decrease; potential increase in admissions
Ienghong et al. 2021	2021	Thailand	Cross-sectional	840	Adult ED	Prehospital POCUS	Standard care	LOS	Slight LOS reduction (159 vs. 165 min), not statistically significant
Duma et al., 2023	2023	France	Prospective QI before–after	N/A	Adult ED	Lean Six Sigma (DMAIC)	Pre-intervention	LOS, door-to-provider time, crowding	Reduced LOS and improved ED flow efficiency

Abbreviations: ED = emergency department; LOS = length of stay; LWBS = left without being seen; CTAS = Canadian Triage and Acuity Scale; WT = waiting time; PIA = physician initial assessment; POCT = point-of-care testing; POCUS = point of care ultrasound; TAT = turnaround time; DMAIC = Define, Measure, Analyze, Improve, Control; NS = not statistically significant

### Summary of study characteristics

The characteristics of the included studies are summarized in Table 2, which presents details of fifteen studies published between 2015 and 2024, conducted across multiple countries including Canada, France, the Netherlands, the United States, Thailand, Brazil, Hong Kong, Australia, and South Africa. Study designs included observational before–after studies, one cluster-randomized trial, simulation models, and quality improvement interventions. Sample sizes varied considerably, ranging from fewer than 800 patients to over 111,000 ED visits. Emergency department (ED) settings included adult-only departments, mixed adult and pediatric EDs, pediatric urgent care units, and dedicated fast-track or critical care zones. Interventions evaluated included fast-track systems, team triage models, comprehensive point-of-care testing (POCT), virtual telehealth tracks, and multifaceted quality improvement bundles. Comparators were typically pre-intervention periods or usual care pathways. Key outcomes included ED LOS, wait times, turnaround time, and crowding indicators such as LWBS rates. Most studies reported improvements in at least one operational metric, with LOS reductions ranging from 9 to 160 min and improvements in flow and efficiency.

**Table 3:** Quality assessment of quantitative studies

Study no.	tool used	Overall risk of bias
Chartier et al., 2015	ROBINS-I	Moderate
Van der Linden et al., 2019	ROBINS-I	Low
Van der Linden et al., 2024	ROBINS-I	Low
Hausfater et al., 2020	Revised Cochrane risk-of-bias tool for randomised trials (RoB 2)	Moderate
Chrusciel et al., 2019	ROBINS-I	Moderate
Copeland et al., 2015	ROBINS-I	Moderate

**Table 4:** Quality assessment of qualitative studies

Study no.	tool used	Aim clearly stated	Methodology appropriate	Data collection	Reflexivity	Overall risk of bias
Kaushik et al. 2018	JBI	Yes	Yes	Clear, multisite & single-site EHR data	Limited	Moderate
Ienghong et al. 2023	JBI	Yes	Yes	Clear, EMS & ED records	Limited	Low
Chaves et al. 2022	CASP	Yes	Yes	Technical visit reports (450)	Partial	High
Goldstein et al. 2018	JBI	Yes	Yes	Observations, staff interviews, workflow documentation	Limited	Moderate
Peng et al. 2020	JBI	Yes	Yes	Historical ED data, clearly described	Limited	Moderate
Duma et al. 2023	JBI	Yes	Yes	ED operational data and process performance indicators; clearly described	Limited	Moderate
Kuo et al. 2018	CASP	Yes	Yes	Historical ED data, simulated patient flows, scenario testing (over 1 million simulated patients across multiple scenarios)	Limited	High
McAvoy et al. 2021	JBI	Yes	Partial	Stakeholder interviews, workshops, site visits; clearly described	Limited	Moderate
Ross et al. 2024	CASP	Yes	Yes	EMR data, process measures (door-to-provider time, length of stay, LWBS rates), staff feedback	Limited	High

### Quality assessment

The risk of bias assessment for the included studies is shown in Tables 3 and 4. A total of 15 studies were assessed using design-appropriate tools, including ROBINS-I for non-randomized studies, the Revised Cochrane Risk of Bias Tool (RoB 2) for randomized controlled trials, and the JBI and CASP critical appraisal checklists for observational and mixed-methods studies.

Across both quantitative and qualitative studies, a total of 3 studies were classified as low risk of bias, 9 as moderate risk, and 2 as high risk, based on design-appropriate appraisal tools.

The most prevalent methodological restrictions were incomplete study protocol reporting, inadequate confounding correction in observational studies, and a lack of information regarding how qualitative and quantitative components are integrated in mixed-methods research. To preserve the validity of the pooled estimates, papers with a high risk of bias were excluded from the meta-analysis.

### Outcomes

**Fig. 2:**
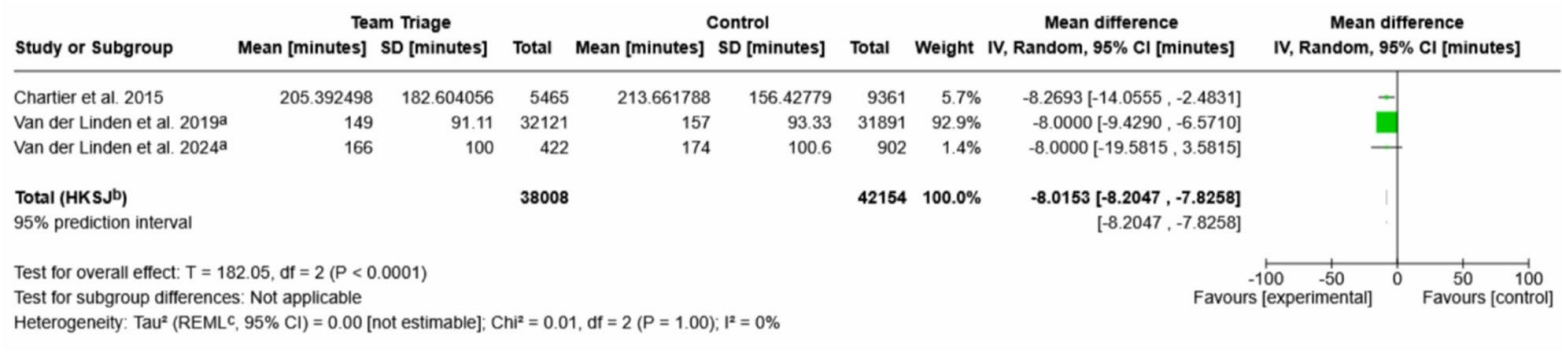
Forest plot for team triage: Team Triage vs. Standard ED

The forest plot of the pooled effect estimates is shown in Fig. 2, illustrating the comparison of wait time outcomes between Team Triage and standard ED care across three studies. The meta-analysis assessed the impact of Team Triage on patient wait time and saw a reduction in mean wait time compared with standard care, with effects varying across study contexts.

Overall, a total of 38,008 patients were included in the Team Triage group and 42,154 patients in the standard ED group. Several studies reported wait times as medians, they were treated as means. The pooled analysis, conducted using a random-effects inverse variance model, yielded a MD of − 8.02 min (95% CI: − 8.20 to − 7.83; *p* < 0.0001), indicating a statistically significant reduction in wait time favoring Team Triage.

Among the individual studies, Chartier et al. (2015) reported a reduction in wait time of − 8.27 min (95% CI: − 14.06 to − 2.48). Van der Linden et al. (2019a), which contributed the largest weight to the analysis, demonstrated a mean difference of − 8.00 min (95% CI: − 9.43 to − 6.57). Similarly, Van der Linden et al. (2024a) reported a mean difference of − 8.00 min, although this estimate did not reach statistical significance (95% CI: − 19.58 to 3.58).

No meaningful heterogeneity was observed across the included studies (I² = 0%, χ² = 0.01, df = 2, *p* = 1.00), and the between-study variance (Tau²) was estimated at 0.00 using the Restricted Maximum Likelihood (REML) method. While no statistical heterogeneity was detected across included studies, the generalizability of Team Triage effects may vary depending on ED setting and implementation context. The 95% prediction interval closely aligned with the pooled estimate, further supporting the robustness of the findings.

**Fig. 3:**
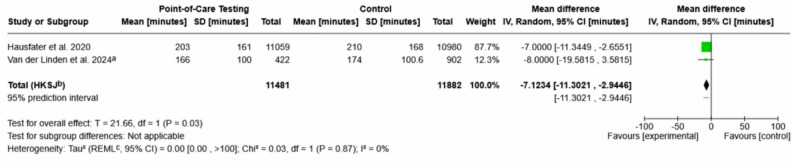
Forest plot for point of care testing: POCT vs. Standard ED

Figure 3 forest plot comparing ED wait time (minutes) between POCT and standard ED care. The effect estimates are expressed as MDs with 95% confidence intervals. The pooled effect was calculated with a random-effects inverse-variance model.

The POCT group had 11,481 patients, while the standard ED group had 11,882 patients. Several studies reported wait times as medians, they were treated as means. The pooled analysis yielded a MD of − 7.12 min (95% CI: − 11.30 to − 2.94; *p* = 0.03), indicating a modest reduction in wait times associated with POCT.

Among the individual studies, Hausfater et al. (2020) reported a reduction in wait time of − 7.00 min (95% CI: − 11.34 to − 2.66) and contributed the majority of the weight to the pooled estimate. Van der Linden et al. (2024a) found a similar point estimate of − 8.00 min; however, it was not statistically significant (95% CI: − 19.58 to 3.58).

No significant heterogeneity was observed between studies (I² = 0%, χ² = 0.03, df = 1, *p* = 0.87), and Tau² was estimated at 0.00 using the REML method. The 95% prediction interval closely aligned with the pooled effect estimate, however, interpretation should consider variability in implementation and study context.

**Fig. 4:**
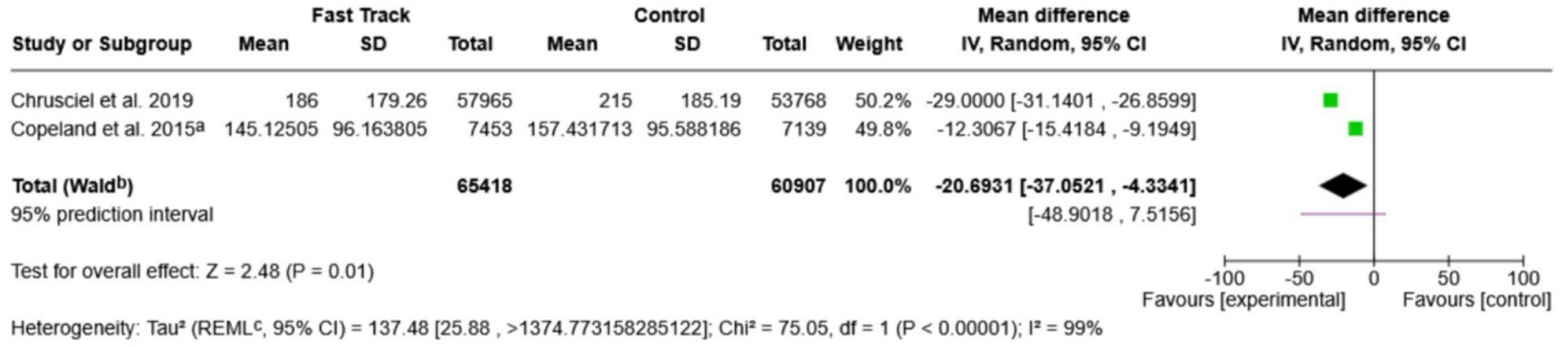
Forest plot for fast track: Fast Track vs. Standard ED

Figure 4 presents a forest plot comparing LOS between Fast-Track interventions and standard ED care across two studies. The meta-analysis showed that Fast-Track implementation decreased patient wait times compared to standard care. Two studies were included, comprising a total of 65,418 patients in the Fast-Track group and 60,907 in the control group. Several studies reported LOS as medians, they were treated as means. Meta-analysis using a random-effects model demonstrated a statistically significant reduction in the LOS favoring the Fast-Track intervention, with a pooled MD of − 20.89 min (95% CI: − 37.05 to − 4.33; *p* = 0.01). The individual study estimates also supported this finding: Chrusciel et al. (2019) reported a MD of − 29.00 min (95% CI: − 31.14 to − 26.86), and Copeland et al. (2015a) reported − 12.31 min (95% CI: − 15.41 to − 9.19).

Substantial heterogeneity was observed across studies, with an I² value of 99%, χ² = 75.05 (df = 1), and *p* < 0.00001. The Tau² was estimated at 137.48 using the REML method. Subgroup analyses by country, study design, and group size did not change the results of the heterogeneity. The small number of studies restricted interpretability. The wide 95% prediction interval (–48.90 to 7.52 min) indicates that the actual impact of Fast-Track interventions may differ significantly between contexts, with some showing little to no benefit. The considerable heterogeneity significantly reduces confidence in the accuracy and generalizability of effect size, thus even though the pooled estimate is statistically significant, it should be considered cautiously.

In conclusion, across all three interventions evaluated: Fast-Track, POCT, and Team Triage meta-analyses revealed varying degrees of effectiveness when compared to standard ED protocols. Among the interventions evaluated, Fast-Track showed the most clinically significant reduction in ED LOS and door-to-provider time compared to standard care. Whereas POCT and Team Triage showed less pronounced, context- dependent effects. Fast-Track pathways showed the largest and most clinically meaningful reduction in LOS, with a pooled MD of − 20.89 min (95% CI: − 37.05 to − 4.33; *p* = 0.01). POCT was associated with a modest pooled reduction in LOS of − 7.12 (95% CI: − 11.30 to − 2.94; *p* = 0.03), while Team Triage was associated with a pooled reduction in wait time of − 8.02 min (95% CI: − 8.20 to − 7.83; *p* < 0.0001), with effects varying by study context. Although individual studies reported larger effects in specific contexts, pooled estimates indicate subtle but consistent benefits. Variability across study settings emphasizes the importance of more research and context-sensitive techniques.

These findings highlight the need for more consistent implementation strategies and further high-quality studies to clarify the effectiveness of POCT and Team Triage models in diverse ED settings.

### Risk of bias assessment (RoB)

All studies included in the meta-analysis were assessed for risk of bias, covering reporting, attrition, performance, detection, selection, and other biases. Tools such as RevMan with the Cochrane RoB 2 and ROBINS-I, along with the JBI Checklists and MMAT for qualitative and mixed-methods studies, were used. Overall, selection bias was minimal, but high-risk concerns arose from inadequate allocation concealment, lack of randomization, and absence of blinding, which increased performance and detection biases. Attrition and selective reporting biases were generally low. Risk of bias varied by study design: observational, retrospective, and before-and-after studies were more prone to selection, detection, and confounding biases. Despite these limitations, the studies provided valuable insights, and no single study was biased in all aspects. Methodological quality differences must be considered when synthesizing evidence to ensure accuracy and fairness.

## Discussion

### Effectiveness of fast-track systems

The results of this systematic review suggest that Fast-Track systems may reduce ED LOS in specific situations; however, the pooled estimate should be interpreted with caution. Even though the meta-analysis showed a mean reduction of − 20.89 min, the highly high heterogeneity (I² = 99%) shows significant variation in effect sizes between studies. This indicates that the benefit is inconsistent and may be influenced by local ED characteristics such as workflow efficiency, staffing, and patient acuity.

Individual studies found reductions in LOS and door-to-provider time, notably in pediatric and low-acuity populations [11, 12, 15, 16], suggesting effectiveness in selected scenarios. The large prediction interval and study diversity, however, imply that the effect’s strength and even direction may vary among institutions. Therefore, Fast-Track showed the largest pooled reduction in LOS among assessed interventions; however, this estimate is limited by high heterogeneity and is not universally applicable.

Fast-track interventions are more effective in high-volume EDs, settings with many low-acuity patients, and institutions with appropriate staffing and infrastructure. These findings emphasize the necessity of context-specific implementation over universal adoption.

### Team triage models

Team Triage, including strategies such as PIT and VVT, consistently decreased wait times ranging from 33% to 77% depending on the scenario [10, 17], with qualitative results showing improvements in patient flow, LOS and wait times [8, 9, 18], the pooled analyses demonstrated a statistically significant but modest overall reduction in wait time, with effectiveness influenced by implementation context [8, 9].

It is important to understand that statistical non-significance does not always imply a lack of therapeutic value. In high-volume or high-acuity emergency departments, even minor savings in triage time can result in significant downstream benefits such as faster patient flow and reduced crowding. Thus, the utility of Team Triage is most likely context-dependent, with the largest potential impact occurring during peak demand periods or at especially congested entry points [8, 9, 17].

### Role of point-of-care testing

POCT was associated with faster diagnostic processes, particularly in high-acuity or complex clinical cases [9, 19, 20]. Its effectiveness, along with Fast-Track pathways, is influenced by hospital capacity and resource allocation. Evidence from Canada suggests feasibility without additional staffing or space [11], while studies from France reported reductions in ED LOS and improvements in care quality within specific institutional contexts [7, 21]. Multimodal interventions and simulation models further demonstrated reductions in overcrowding during peak hours [9, 22, 23].

Quantitative analyses showed modest reductions in wait times for Team Triage (8.02 min) and POCT (7.12 min) compared to standard care. Larger reductions in LOS were observed in individual studies and specific clinical settings, but these were not reflected in the pooled meta-analytic estimate [24]. Although pooled results may lack consistent significance, POCT remains useful, especially where diagnostic delays limit decision-making. Its benefits are most noticeable in high-acuity or complex cases.

Overall, Fast-Track provides more consistent benefits, while POCT and Team Triage show context-dependent improvements influenced by local resources and implementation.

### Sources of heterogeneity

Pooled analyses for fast-track interventions showed significant heterogeneity. This variability likely reflects differences in patient acuity and baseline LOS across EDs. Patient flow may have been impacted by variability in structure of healthcare system, including referrals, funding, and access to healthcare. Institutional differences such as staffing, bed capacity, and admission processes also contribute to heterogeneity.

Subgroup analyses based on geographical location, ED type, patient volume, and acuity were conducted. However, no significant changes were observed, and heterogeneity remained high.

Crucially, the observed degree of heterogeneity (I² = 99%) significantly restricts the pooled estimate’s robustness and generalizability, suggesting that the actual impact of Fast-Track interventions is highly context dependent. According to the Cochrane Handbook for Systematic Reviews of Interventions [25], variability is expected in complex health interventions. Therefore, results should be interpreted considering institutional ED characteristics.

### Mechanism of impact

These interventions improve ED workflow by targeting specific inefficiencies. Fast-Track systems reallocate resources by directing low-acuity patients to their selected areas, allowing staff and treatment areas to focus on higher-acuity cases [26]. Team Triage interventions evaluate each case initially, supporting quick decision-making and smoothing patient flow reducing delays at entry points [27]. POCT minimizes diagnostic delays via rapid bedside testing [28]. Together, they highlight the importance of tailored strategies for each ED.

### Intervention impact across populations

Fast-Track systems were primarily effective for non-urgent, low-acuity patients [10, 12, 15, 16, 18], while POCT demonstrated utility in both low and high-acuity patients by speeding diagnostics [23]. Team Triage interventions, were applied to general ED populations, addressing a broad range of presentations [8, 19, 20, 22, 24]. Additionally, specific interventions targeted pediatric populations [10, 12] and ambulance patients [11], highlighting the diversity of patient groups considered in these studies.

### Demographic characteristics of included studies

Pediatric populations were specifically addressed in one study [12], while a study included both pediatric and adult care [10]. Most studies focused on adult populations [7, 21]. Demographic differences among providers were explored [13], though effectiveness limitations reduced insight from these analyses. No studies exclusively targeted elderly, gender-specific or disadvantaged subgroups, highlighting the need for further research across a broader range of populations.

### Geographical variations and healthcare

Geographical context further influenced the implementation. Most studies were conducted in high-resource settings, such as Canada [7, 17, 18], the United States [10, 12, 13, 22], and European countries like the Netherlands and Italy [8, 9, 21, 23, 24]. These regions benefit from strong healthcare systems that support intervention integration. In contrast, studies in Asia [16, 19], South Africa and Brazil [19, 20] highlighted the unique challenges faced in low-resource settings, including urban-rural disparities and overcrowded public healthcare systems.

Low- and middle-income countries may benefit more from basic infrastructure improvements before adopting resource-intensive interventions [29]. Cost-effectiveness and scalability remain key concerns [30], emphasizing context-specific adaptation.

#### Regional bias and study distribution

Although multiple regions were included, geographical bias was evident. Most studies were from North America and Europe, with fewer from Africa and South America [7–10, 12, 13, 16, 17, 20, 23, 24]. This imbalance limits generalizability due to differences in healthcare systems and resources [16, 19, 20]. Future research should focus on underrepresented regions for a more comprehensive understanding [29, 30].

### Strengths of this review

This study has several strengths. First, it followed PRISMA guidelines to ensure a comprehensive process. Second, Rayyan was used for screening by multiple reviewers, enhancing consistency and reducing bias. The process used clear inclusion and exclusion criteria focusing on hospital-based ED interventions. The study addresses a global issue ED overcrowding making findings highly relevant. Finally, a rigorous methodology using multiple databases and tools strengthened the reliability of results.

### Limitations of the evidence base

This review faced several limitations. Included studies used diverse methodologies, making synthesis difficult. Differences in data quality and reporting, especially for measures like LOS and wait times, further added to this challenge. Generalizability was also limited because many studies were single-centre investigations, often in rural or low-resource settings where interventions may function differently [23, 25]. Study size varied widely as well, from small cohorts to analyses of millions of patient encounters [15], reducing consistency across the evidence base. Moreover, several studies did not clearly report intervention duration, making it hard to assess the long-term sustainability of these strategies [16, 18, 20, 23]. Several studies also lacked clear definitions of key outcomes, such as wait time and ED LOS. In these cases, outcome classifications were deduced based on standard ED workflows, which may introduce a risk of misclassification bias.

### Literature gaps and research challenges

Literature gaps were evident in the lack of knowledge about the scalability and long-term effectiveness of interventions such as POCT [9, 19, 22] or Team Triage [8–10, 17] in diverse healthcare systems. For instance, the performance of these interventions under resource constraints or during pandemics remains unexplored. Moderate levels of bias, as revealed in the risk assessments, stemmed from issues like selection bias and data quality limitations, compounded by external factors such as the COVID-19 pandemic and infrastructural challenges [18, 23]. Despite these limitations, the review provides valuable insights into ED interventions, emphasizing the importance of contextually appropriate and evidence-based interpretations [11, 12, 15, 22].

### Implications for clinical practice

The results of this systematic review have far-reaching implications for clinical practice, policymaking, and future research. Interventions showed improvements in wait times and LOS [9, 10, 17, 22], but did not consistently reduce overall ED overcrowding [20]. Team Triage models improved flow and reduced delays, while POCT accelerated diagnostics in high-acuity cases. These interventions prove to be feasible to execute and offer benefits such as improved patient satisfaction, increased efficiency during peak demand periods, and reduced provider burnout [20].

Clinicians should interpret these findings with an understanding that modest pooled effect sizes do not necessarily imply a lack of usefulness. The therapeutic impact of interventions such as Team Triage and POCT is heavily influenced by local emergency department features such as patient volume, acuity, staffing, and congestion [9, 17, 20]. As operational tools, these interventions may provide targeted benefits when used strategically rather than as universal solutions across all settings [20].

### Implications for policy

From a policy perspective, the findings underscore the need for directed funding toward proven strategies especially Fast-Track systems [8–10]. Addressing disparities between urban and rural settings is essential, as many of these interventions remain underutilized in resource-limited areas [16, 23]. Establishing universal policies for ED management could further optimize patient outcomes [8, 9].

### Future research directions

Future studies should focus on low-cost adaptations and long-term outcomes. Multi-center studies with robust methodologies can generate globally relevant evidence, Multi-center and mixed-methods research can improve generalizability and understanding. Research should also consider pandemics and resource limitations when designing interventions. Addressing these gaps will help reduce ED overcrowding and improve care quality [9, 20].

## Limitations

This review has several methodological and contextual limitations. Some studies reported LOS as medians rather than means; because patient-level data were unavailable and RevMan Web does not support pooling medians, medians were assumed to approximate means. In ED settings, LOS is often right-skewed; therefore, medians are usually lower than means, which may underestimate the true average LOS and intervention effects. However, depending on variability, both underestimation and overestimation are possible. The exact degree of bias cannot be determined without raw data; therefore, pooled estimates should be interpreted with caution [25].

Variability in outcome definitions (e.g., wait time and ED LOS) and inclusion of different Team Triage models contributed to heterogeneity. Significant statistical heterogeneity was identified, especially for Fast-Track interventions (I² = 99%), limiting confidence in the precision and generalizability of pooled results.

Sensitivity analyses showed consistent effect direction, suggesting no single study strongly influenced results. Minor variations in heterogeneity and effect sizes were observed, indicating that assumptions such as treating medians as means may still affect pooled estimates; therefore, results should be interpreted with caution [25].

Most studies were conducted in high-resource settings, limiting generalizability to low-resource environments. Finally, the moderate risk of bias in many included studies and substantial heterogeneity, particularly for Fast-Track systems, reduce confidence in pooled estimates.

**Table 5:** Overall certainty of evidence for each intervention

Intervention	No. of studies	Risk of bias	Certainty of evidence	Justification
Fast Track	2	Moderate	Low-Moderate	Effect direction is consistent despite high heterogeneity; moderate risk of bias slightly reduces certainty
Team Triage	3	Moderate	Low	Modest effect; Moderate risk of bias
POCT	2	Moderate	Low	Modest effect; Moderate risk of bias

Table 5 summarizes the certainty of evidence, highlighting feasible interventions while emphasizing the need for further research.

Future research should evaluate these interventions across diverse healthcare systems and assess their cost-effectiveness to better inform policy and resource allocation.

## Conclusion

This systematic review and meta-analysis integrates evidence on interventions aimed at reducing ED overcrowding and improving patient outcomes and operational efficiency. Using a mixed-methods approach allowed comprehensive evaluation through the integration of quantitative and qualitative data. Key findings highlight the role of Fast-Track systems, POCT, and Team Triage in reducing ED outcomes such as LOS, wait times, and discharge times. Fast-Track interventions demonstrated the most pooled reductions in LOS, but with substantial heterogeneity, reducing the confidence in the accuracy and generalizability of effect estimates. Fast track interventions exhibit moderate risk of bias and low-moderate certainty of evidence, while POCT and Team Triage showed potential benefit but with moderate risk of bias and low certainty of evidence. However, these findings should be interpreted cautiously due to variability across studies and limited statistical evidence. Overall, these findings provide insight into evidence-based strategies for optimizing ED performance and may guide emergency physicians and hospital administrators seeking sustainable solutions to ED overcrowding.

## Recommendations

Based on our findings, Fast-Track systems consistently demonstrated reductions in ED wait times and LOS, particularly for low-acuity patients, suggesting strong potential for improving operational outcomes.

Given the low–moderate certainty of evidence, Fast-Track interventions can be considered for implementation in appropriate settings, with consideration of local resources and patient characteristics.

Team Triage and POCT showed low certainty of evidence, indicating the need for careful, context-specific implementation.

Due to the low certainty of evidence, these interventions should be applied cautiously, and preferably within settings that allow monitoring and evaluation of outcomes.

Since the majority of included studies were conducted in high-resourced healthcare systems, generalizability to low-resource settings may be limited.

## Electronic Supplementary Material

Below is the link to the electronic supplementary material.

Supplementary Material 1

## Data Availability

Data used in this study are available from the corresponding author upon reasonable request.
