# Supplementary material for: Interventions to reduce emergency department overcrowding and their effects on patient outcomes: a mixed-methods systematic review and meta-analysis
Source: BMC Emerg Med. 2026 Apr 25;26:169. doi: 10.1186/s12873-026-01587-8 (PMC13255496; doi:10.1186/s12873-026-01587-8)
Supplement: Supplementary file 1 — Supplementary Material 1 [file 12873_2026_1587_MOESM1_ESM.pdf]

Supplementary table S1: Full Search Strategies for All Databases

Database	Date Searched	Search String Used
PubMed		"Emergency Service, Hospital"[MeSH] OR "Emergency Service, Hospital, Nursing"[MeSH] OR "Emergency department"[tiab] OR "Emergency room"[tiab] OR "Emergency unit"[tiab]) AND ("Length of Stay"[MeSH] OR "waiting time"[MeSH] OR "wait time"[tiab] OR "length of stay"[tiab] OR "door to provider"[tiab] OR "time to triage"[tiab] OR "time to disposition"[tiab]) AND ("Triage"[MeSH] OR "Triage, Emergency"[MeSH] OR "fast track"[tiab] OR "fast-track"[tiab] OR "physician in triage"[tiab] OR "team triage"[tiab] OR "tele-triage"[tiab]) OR ("Point-of-Care Systems"[MeSH] OR "Point-of-Care Testing"[tiab] OR "POCT"[tiab] OR "bedside testing"[tiab]) OR ("Decision Support Systems, Clinical"[MeSH] OR "Clinical Decision Support System"[tiab] OR "CDSS"[tiab])
Google Scholar		"fast track emergency department length of stay" OR "emergency room and length of stay" OR "point of care testing in emergency room and length of stay" OR "team triage emergency department" OR "POCT emergency department wait time"
ScienceDirect		("Emergency department" AND "fast track") AND ("length of stay" OR "wait time" OR "patient flow")

Supplementary Table S2. Keyword Groups and Synonyms Used

Concept	MeSH Terms	Free-text Keywords
Emergency Department	Emergency Service, Hospital	emergency department, emergency room, emergency unit
Wait Time / LOS	Length of Stay; Waiting Time	wait time, ED LOS, patient flow, door-to-provider
Interventions	Triage; Point-of-Care Systems	fast track, team triage, physician in triage, tele-triage, POCT, bedside testing
Decision Support	Decision Support Systems, Clinical	CDSS, clinical decision tool
